# Inpatient direct oral challenge for sulfa antibiotic allergy: improving care in immunocompromised hosts

**DOI:** 10.1017/ice.2026.10400

**Published:** 2026-04

**Authors:** Elise A. Mitri, Sara Vogrin, Rebecca Hall, Ronald Ma, Gemma K. Reynolds, Jason A. Trubiano

**Affiliations:** 1 Department of Infectious Diseases, The Peter Doherty Institute for Infection and Immunity, University of Melbourne, Melbourne, VIC, Australia; 2 Centre for Antibiotic Allergy and Research, Department of Infectious Diseases and Immunology, Austin Health, Heidelberg, VIC, Australia; 3 National Allergy Centre of Excellence (NACE), Parkville, VIC, Australia; 4 Department of Finance, Austin Health, Heidelberg, VIC, Australia

## Abstract

In this prospective cohort study, trimethoprim-sulfamethoxazole direct oral challenge (DOC) for hospitalized adults reporting a low-risk sulfa antibiotic allergy was safe with 75/76 (99%) inpatients delabeled. Within 90-days of DOC, immunocompromised patients were more likely to receive trimethoprim-sulfamethoxazole, compared with non-immunocompromised patients (adjusted OR 5.6 95% CI 1.3, 23.0).

## Introduction

Over the last decade, direct oral challenge (DOC) without prior skin-testing has been increasingly utilized as the standard of care for delabeling patients with a low-risk penicillin allergy. The incorporation of penicillin DOC for low-risk allergy phenotypes into Antimicrobial Stewardship (AMS) interventions has significantly improved antibiotic appropriateness, especially in immunocompromised hosts.^1^ This has been enabled by the derivation, validation and implementation of penicillin allergy risk assessment tools and the non-inferiority of DOC compared to traditional skin-testing.^2,3^ Similarly, risk assessment and DOC have been successfully applied in the outpatient setting to low-risk sulfa antibiotic allergies.^4,5^ In efforts to improve timely access to sulfa antibiotics for hospitalized patients who are more medically complex than a traditional outpatient population, we sought to evaluate the safety, effectiveness and healthcare savings of low-risk sulfa antibiotic allergy delabeling via DOC in the inpatient setting, with a focus on the immunocompromised host.

## Methods

### Study design, setting and intervention

A prospective cohort study was undertaken at Austin Health, a tertiary referral hospital in Melbourne, Australia. Inpatients on acute wards who reported a sulfa or trimethoprim-sulfamethoxazole allergy were identified by the multidisciplinary AMS-Allergy service and assessed utilizing the Antibiotic Allergy Assessment Tool (AAAT)^6^ and when derived in 2023, the SULF-FAST clinical decision rule (Figure S1).^7^ The AAAT was used to characterize the sulfa allergy phenotype, and the SULF-FAST clinical decision rule was applied to determine the risk of a positive DOC. Inpatients with a low-risk assessment per the AAAT and/or SULF-FAST score<3 were offered a single-dose trimethoprim-sulfamethoxazole DOC (Appendix S1). Patients who were hemodynamically unstable, unable to provide consent, or concurrently prescribed antihistamines or prednisolone (or equivalent corticosteroid)>25 mg daily were excluded from inpatient DOC. The prednisolone threshold of ≤25 mg daily was chosen as there are limited international consensus recommendations for the maximum corticosteroid threshold at which antibiotic challenge should occur, and this study aimed to improve imminent access to first-line antibiotics, particularly in immunocompromised patients with indeterminate plans for corticosteroid weaning.

Following DOC, the hospital medical record was audited for trimethoprim-sulfamethoxazole utilization within 90-days and relabeling of trimethoprim-sulfamethoxazole allergy was audited at 12-months.

Immunocompromised hosts were defined as i) hemotological or solid organ malignancy, ii) solid organ transplant, or iii) other immunocompromise; autoimmune or connective tissue disorder, human immunodeficiency virus, hemodialysis, end-stage chronic liver disease, splenectomy or receiving prednisolone (or equivalent)>10mg/day for more than one month.

Data were managed and stored using REDCap electronic data capture tools and Ethics approval was obtained from the Austin Health Research Ethics Committee (47585/Austin-2018).

The outcomes were (i) safety; the proportion of inpatients with a low-risk sulfa allergy that were delabeled following DOC, (ii) effectiveness; the proportion of patients who utilized trimethoprim-sulfamethoxazole within 90-days of DOC, and (iii) healthcare savings; the comparative healthcare costs of inpatient DOC and subsequent trimethoprim-sulfamethoxazole use for immunocompromised patients requiring *Pneumocystis jirovecii* pneumonia (PJP) prophylaxis versus alternative PJP prophylaxis therapy.

### Statistical analysis

Characteristics were compared between immunocompromised and non-immunocompromised groups using Fisher’s Exact or rank sum test. Logistic regression was used to evaluate utilization of trimethoprim-sulfamethoxazole post-DOC between immunocompromised and non-immunocompromised groups.

## Results

Between 23 March 2022 and 14 May 2025, 221 inpatients with a reported trimethoprim-sulfamethoxazole or sulfa antibiotic allergy were assessed, of which 118 (53%) were low-risk on the AAAT and 140 (63%) had a SULF-FAST score<3.

In total, 76 (34%) inpatients underwent DOC, of which 30 (40%) were immunocompromised hosts (Table 1). Of 30 inpatients in the immunocompromised host group, 18 (60%) had a hematological or solid organ malignancy, six (20%) were solid organ transplant recipients, and six (20%) were immunocompromised for other reasons (Table S1). Of 76 inpatient trimethoprim-sulfamethoxazole DOC performed, 75 (99%) were delabeled. One non-immunocompromised patient experienced a delayed exanthem that was managed in the outpatient setting with an antihistamine.

**Table 1.** Cohort and allergy phenotype characteristics of immunocompromised host vs non-immunocompromised host inpatients undergoing trimethoprim-sulfamethoxazole direct oral challenge

A. Cohort characteristics	Overall (*N* = 76)	Immunocompromised host^*†*^ (*N* = 30)	Non-immunocompromised host (N = 46)	*P* value
*Sex*				0.64
Female	44 (58%)	16 (53%)	28 (61%)	
Male	32 (42%)	14 (47%)	18 (39%)	
Age (years), median (IQR)	67 (58.5, 77)	69 (61, 77)	67 (55, 77)	0.46
*Ethnicity*				>0.99
White	70 (92%)	28 (93%)	42 (91%)	
Other	6 (8%)	2 (7%)	4 (9%)	
*Admitting Unit*				0.006
Medical Immunocompromised host^*‡*^	6 (8%)	6 (20%)	0 (0%)	
Medical other	25 (33%)	6 (20%)	19 (41%)	
Surgical	28 (37%)	10 (33%)	18 (39%)	
Other	17 (22%)	8 (27%)	9 (20%)	
Charlson comorbidity index, median (IQR)	4 (3, 6.5)	6.5 (5, 9)	4 (2, 5)	<0.001
*Infective diagnosis on admission*				0.16
No	10 (13%)	2 (7%)	8 (17%)	
Yes	24 (32%)	13 (43%)	11 (24%)	
Unknown, pending work-up	42 (55%)	15 (50%)	27 (59%)	
Non-antibiotic drug allergy	33 (43%)	16 (53%)	17 (37%)	0.16
*Number of antibiotic allergies*				0.46
Single	51 (67%)	22 (73%)	29 (63%)	
Multiple	25 (33%)	8 (27%)	17 (37%)	
Concurrent penicillin allergy	16 (21%)	4 (13%)	12 (26%)	0.18
Non-penicillin, beta-lactam allergy	5 (7%)	2 (7%)	3 (7%)	0.98
B. Allergy phenotype characteristics	Overall (*N* = 76)	Immunocompromised host^*†*^ (*N* = 30)	Non-immunocompromised host (*N* = 46)	*P* value
*Implicated antibiotic name*				0.81
Trimethoprim-sulfamethoxazole	43 (57%)	16 (53%)	27 (59%)	
Sulfa antibiotic unspecified	33 (43%)	14 (47%)	19 (41%)	
*Antibiotic Allergy Assessment Tool (AAAT)* ^*§*^ *Risk assessment*				0.036
White—very low-risk	6 (8%)	5 (17%)	1 (2%)	
Green—low-risk	67 (88%)	25 (83%)	42 (91%)	
Orange—moderate-risk	3 (4%)	0 (0%)	3 (7%)	
*SULF-FAST score*				0.39
0	26 (34%)	11 (37%)	15 (33%)	
1	49 (65%)	18 (60%)	31 (67%)	
2	1 (1%)	1 (3%)	0 (0%)	
Reaction description*				
Childhood exanthem	17 (22%)	3 (10%)	14 (30%)	0.037
Immediate diffuse rash	1 (1%)	0 (0%)	1 (2%)	0.42
Diffuse rash >5–10 years ago	28 (37%)	15 (50%)	13 (28%)	0.055
Angioedema	0 (0%)			
Generalized swelling (not angioedema)	1 (1%)	0 (0%)	1 (2%)	0.42
Urticaria	7 (9%)	2 (7%)	5 (11%)	0.54
Anaphylaxis or unexplained collapse	0 (0%)			
Unknown reaction >5–10 years prior	14 (18%)	5 (17%)	9 (20%)	0.75
Gastrointestinal symptoms	8 (11%)	5 (17%)	3 (7%)	0.16
Other	8 (11%)	3 (10%)	5 (11%)	0.90
*Treatment required for reaction*				0.63
Yes	6 (8%)	3 (10%)	3 (7%)	
No	27 (36%)	12 (40%)	15 (33%)	
Unknown	43 (57%)	15 (50%)	28 (61%)	
*Hospitalized following reaction*				0.45
Yes	1 (1%)	0 (0%)	1 (2%)	
No	72 (95%)	28 (93%)	44 (96%)	
Unknown	3 (4%)	2 (7%)	1 (2%)	

IQR: interquartile range

† Immunocompromised hosts—solid organ transplant recipient, hematological or solid organ malignancy, autoimmune or connective tissue disorder, human immunodeficiency virus, hemodialysis, end-stage chronic liver disease, splenectomy or prednisolone (or equivalent) use>10mg/day for 1 month.

‡ Medical Immunocompromised host: Oncology, Hematology, Liver transplant.

§ As per previously published AAAT.

* Patients may report more than one reaction description.

Seventeen (22%) patients utilized trimethoprim-sulfamethoxazole within 90-days of DOC; 10 (13%) for therapeutic indications and 7 (9%) for PJP prophylaxis. There was greater use of subsequent trimethoprim-sulfamethoxazole (prophylaxis or treatment) in the immunocompromised host cohort (40% vs 11%, *P* = 0.004). After adjusting for age, sex, and Charlson Comorbidity Index, immunocompromise status was a predictor of increased use of trimethoprim-sulfamethoxazole post-DOC (adjusted OR 5.6 95% CI 1.3, 23.0) (Table 2). The outcomes of 30 immunocompromised hosts who underwent inpatient DOC, stratified by sub-group, are in Table S2.

**Table 2.** Outcomes of immunocompromised host vs non-immunocompromised host inpatients undergoing trimethoprim-sulfamethoxazole direct oral challenge

Outcomes post-DOC	Overall (*N* = 76)	Immunocompromised host^*†*^ (*N* = 30)	Non-immunocompromised host (*N* = 46)	aOR (95% CI)*, *P* value
Delabeled post-DOC	75 (99%)	30 (100%)	45 (98%)	
Trimethoprim-sulfamethoxazole use postDOC (at 90-days)	17 (22%)	12 (40%)	5 (11%)	5.6 (1.3, 23.0), 0.004
*Indication for trimethoprim-sulfamethoxazole use post-DOC (within 90-days)*				
Not used	59 (78%)	18 (60%)	41 (89%)	<0.001
Prophylaxis	7 (9%)	7 (23%)	0 (0%)	
Therapeutic	10 (13%)	5 (17%)	5 (11%)	
*Indication for therapeutic trimethoprim-sulfamethoxazole (within 90-days)*				
Gram-negative bacteremia	5 (7%)	3 (10%)	2 (4%)	
Intra-abdominal infection	2 (3%)	1 (3%)	1 (2%)	
Skin and soft tissue infection	1 (1%)	1 (3%)	0 (0%)	
Pyelonephritis	2 (3%)	0 (0%)	2 (4%)	
Relabeled post-DOC (within 12-months)	1 (1%)^**^	0 (0%)	1 (2%)	
Mortality at 90-days postDOC	2 (3%)	2 (7%)	0 (0%)	n/a, 0.15

*DOC: direct oral challenge, aOR: adjusted odds ratio.*

*†Immunocompromised hosts - transplant recipient, hematological or solid organ malignancy, autoimmune or connective tissue disorder, human immunodeficiency virus, hemodialysis, end-stage chronic liver disease, splenectomy or prednisolone (or equivalent) use>10mg/day for 1 month.*

*Univariable logistic regression or Fisher’s Exact.

**Delayed positive direct oral challenge, as described.

Aside from the single positive challenge event, no patients had their allergy label reinstated in the medical record at 12-months post-DOC. Mortality, unrelated to DOC, occurred in 2 (3%) patients within 90-days of evaluation (Table 2). Of note, four (5%) inpatients also underwent penicillin allergy delabeling via DOC during the same hospital admission.

Of 145 patients who did not undergo inpatient DOC, 22 (15%) were directly delabeled based on history alone, 35 (24%) were medically unstable precluding inpatient DOC, and 10 (7%) were discharged prior to DOC. In total, 26 (18%) patients declined testing in the inpatient setting, most with a preference to defer testing to post-discharge, and a smaller proportion declining any future evaluation. Further, 52 (36%) patients were assessed as moderate-high risk phenotypes and inappropriate for inpatient DOC, however were offered evaluation in the outpatient antibiotic allergy clinic.

In the Australian context, for immunocompromised inpatients requiring PJP prophylaxis, use of DOC to delabel low-risk sulfa antibiotic allergy, and enable first-line trimethoprim-sulfamethoxazole therapy in lieu of inhaled pentamidine, the projected healthcare savings were estimated at $416.94 AUD per delabeled patient for 6-months of PJP prophylaxis (Table S3).

## Discussion

This study is one of the first to examine the utility of trimethoprim-sulfamethoxazole DOC for inpatients with a low-risk sulfa antibiotic allergy, facilitating access to sulfa antibiotics where an acute therapeutic need exists, and eliminating delays to accessing outpatient testing. The approach appears safe and led to appropriate trimethoprim-sulfamethoxazole utilization for both prophylaxis and treatment, especially in immunocompromised patients. Although limited by its single regional experience, small study numbers, and predominance of patients with a SULF-FAST score of 0–1, the findings are supported by the prior success of a similar penicillin allergy delabeling program,^1^ external international validation of the SULF-FAST clinical decision rule,^8^ and known similar immunological patterns showing loss of immunogenicity over time for both penicillin and sulfa antibiotic allergies.^4^ The use of inpatient trimethoprim-sulfamethoxazole DOC to evaluate low-risk sulfa antibiotic allergy, particularly in patients with remote (>5 years) non-anaphylactic, benign cutaneous reactions, negates the requirement for time- and resource-intensive desensitization protocols, and further supports sulfonamide allergy testing recommendations from the American Academy of Asthma Allergy and Immunology.^9^ In addition to the healthcare savings described, further economic benefits may be achieved by implementing DOC for low-risk sulfa antibiotic allergy and reserving the use of desensitization for patients with a convincing history of anaphylaxis.^9^ However, unlike penicillin allergy, trimethoprim-sulfamethoxazole is a drug more commonly associated with severe cutaneous adverse reactions, and education around risk assessment, including identifying non-cutaneous features of severe cutaneous adverse reactions such as organ dysfunction, is required to ensure only low-risk phenotypes undergo DOC. Nonetheless, this study highlights the promise of inpatient sulfa antibiotic allergy delabeling via DOC for low-risk phenotypes, addressing an identified need in immunocompromised hosts where trimethoprim-sulfamethoxazole is a preferred prophylaxis and treatment strategy, and its avoidance is associated with inferior infection outcomes.^10^ Further studies demonstrating the safety, effectiveness, and implementation of inpatient trimethoprim-sulfamethoxazole DOC, especially for immunocompromised hosts, will help confirm this strategy as a novel AMS tool.

## Supporting information

Mitri et al. supplementary material Mitri et al. supplementary material

## Supplementary material

The supplementary material for this article can be found at https://doi.org/10.1017/ice.2026.10400
