# Supplementary material for: Inpatient direct oral challenge for sulfa antibiotic allergy: improving care in immunocompromised hosts
Source: Infect Control Hosp Epidemiol. 2026 Feb 2;47(4):409–13. doi: 10.1017/ice.2026.10400 (PMC13216812; doi:10.1017/ice.2026.10400)
Supplement: Mitri et al. supplementary material [file S0899823X26104000sup001.docx]

**Supplementary Appendices:** Inpatient Direct Oral Challenge for Sulfa Antibiotic Allergy: Improving care in immunocompromised hosts

TABLE OF CONTENTS

[Figure S1: SULF-FAST Clinical Decision Rule, adapted from the PEN-FAST Clinical Decision Rule 2](#_Toc218544552)

[Appendix S1: Approach to inpatient trimethoprim-sulfamethoxazole direct oral challenge 3](#_Toc218544553)

[Table S1: Cohort and Allergy phenotype characteristics of 30 immunocompromised hosts undergoing trimethoprim-sulfamethoxazole direct oral challenge, stratified by immunocompromised host category. 4](#_Toc218544554)

[Table S2: Outcomes of 30 immunocompromised hosts undergoing inpatient trimethoprim-sulfamethoxazole direct oral challenge. 6](#_Toc218544555)

[Table S3: Estimated healthcare cost savings for utilisation of inpatient trimethoprim-sulfamethoxazole direct oral challenge for immunocompromised patients requiring Pneumocystis jirovecii pneumonia prophylaxis in the Australian setting† 7](#_Toc218544556)

[References 8](#_Toc218544557)

# Figure S1: SULF-FAST Clinical Decision Rule, adapted from the PEN-FAST Clinical Decision Rule

**Sulfa antibiotic allergy reported by patient**

**SULF-FAST Clinical Decision Rule**

**Assessment:**

- **Five years or less since the reaction (2 points)**
- **Anaphylaxis or angioedema (2 points)**

  OR

- **Severe cutaneous adverse reaction (2 points)**
- **Treatment required for reaction (1 point)**

**Interpretation:**

Low-risk sulfa antibiotic allergy; SULF-FAST score < 3

Negative Predictive Value:

98.1% (95% CI 93.5-99.8) – Australian cohort

95.5% (95% CI 91.4-98.1) – USA cohort^2^

Yes

**Penicillin allergy reported by patient**

Yes

**PEN-FAST Clinical Decision Rule**

**Assessment:**

- **Five years or less since the reaction (2 points)**
- **Anaphylaxis or angioedema (2 points)**

  OR

- **Severe cutaneous adverse reaction (2 points)**
- **Treatment required for reaction (1 point)**

**Interpretation:**

Low-risk penicillin allergy; PEN-FAST score < 3

Negative Predictive Value:

96.3% (95% CI 94.1-97.8)^1^

# Appendix S1: Approach to inpatient trimethoprim-sulfamethoxazole direct oral challenge

The health service antimicrobial stewardship allergy ward round and approach to inpatient penicillin allergy delabeling has previously been described.^3^ A similar approach was employed for implementing low-risk sulfa antibiotic allergy delabeling via direct oral challenge in the inpatient setting.

In brief, following sulfa antibiotic allergy assessment, patients with a low-risk sulfa antibiotic allergy (‘green’ on the Antibiotic Allergy Assessment Tool and/or SULF-FAST score <3) were counselled on inpatient sulfa antibiotic allergy testing via direct oral challenge. If accepted by the patient, written informed consent was obtained prior to prescription of a single oral dose of 80/400 mg trimethoprim-sulfamethoxazole using an electronic medical record order set. Baseline observations and an additional set of observations were performed at 30- and 60-minutes post-challenge.

If there was no immediate adverse event during the 60-minute monitoring period, the sulfa antibiotic allergy label was removed, the patient was counseled to monitor for and report any delayed adverse events, and contact details of the Antimicrobial Stewardship (AMS)-Allergy team were provided. If a therapeutic sulfa antibiotic prescription was required, this was commenced following a negative direct oral challenge after the 60-minute monitoring period. Immediate or delayed adverse events occurring either during the inpatient admission or following discharge were managed by the AMS Allergy team.

The patient’s medical record was updated with the outcome of direct oral challenge, and the hospital treating team and patient’s primary care provider were notified in writing.

# Table S1: Cohort and Allergy phenotype characteristics of 30 immunocompromised hosts undergoing trimethoprim-sulfamethoxazole direct oral challenge, stratified by immunocompromised host category.

| **A. Immunocompromised host cohort characteristics** | **Hematological or solid organ malignancy**  **(n=18)** | **Solid organ transplant**  **(n=6)** | **Other*†***  **(n=6)** | **P value** |
| --- | --- | --- | --- | --- |
| *Sex* | | | |  |
| Female | 7 (39%) | 4 (67%) | 5 (83%) | 0.18 |
| Male | 11 (61%) | 2 (33%) | 1 (17%) |  |
| Age (years), median (IQR) | 73.5 (66, 81) | 64.5 (61, 69) | 56 (36, 77) | 0.062 |
| *Ethnicity* | | | |  |
| White | 16 (89%) | 6 (100%) | 6 (100%) | 1.00 |
| Other | 2 (11%) | 0 (0%) | 0 (0%) |  |
| Charlson comorbidity index, median (IQR) | 8 (6, 9) | 5.5 (4, 7) | 4.5 (2, 5) | 0.003 |
| *Infective diagnosis on admission* | | | |  |
| No | 2 (11%) | 0 (0%) | 0 (0%) | 0.96 |
| Yes | 8 (44%) | 3 (50%) | 2 (33%) |  |
| Unknown, pending work-up | 8 (44%) | 3 (50%) | 4 (67%) |  |
| *Taking concurrent prednisolone (or equivalent systemic corticosteroid) on day of DOC* | | | | |
| Yes^*^ | 2 (11%) | 4 (67%) | 1 (17%) | 0.019 |
| Non-antibiotic drug allergy | 6 (33%) | 6 (100%) | 4 (67%) | 0.015 |
| *Number of antibiotic allergies* | | | |  |
| Single | 13 (72%) | 6 (100%) | 3 (50%) | 0.14 |
| Multiple | 5 (28%) | 0 (0%) | 3 (50%) |  |
| Concurrent penicillin allergy | 3 (17%) | 0 (0%) | 1 (17%) | 0.80 |
| Non-penicillin, beta-lactam allergy | 0 (0%) | 0 (0%) | 2 (33%) | 0.069 |
| **B. Allergy phenotype characteristics** | **Hematological or solid organ malignancy**  **(n=18)** | **Solid organ transplant**  **(n=6)** | **Other*†***  **(n=6)** | **P value** |
| *Implicated antibiotic name* | | | |  |
| Trimethoprim-sulfamethoxazole | 7 (39%) | 4 (67%) | 5 (83%) | 0.18 |
| Sulfa antibiotic unspecified | 11 (61%) | 2 (33%) | 1 (17%) |  |
| *Antibiotic Allergy Assessment Tool (AAAT)^§^ Risk assessment* | | | |  |
| White – very low risk | 3 (17%) | 2 (33%) | 0 (0%) | 0.34 |
| Green - low risk | 15 (83%) | 4 (67%) | 6 (100%) |  |
| Orange - moderate risk | 0 (%) | 0 (%) | 0 (%) |  |
| *SULF-FAST score* | | | |  |
| 0 | 9 (50%) | 2 (33%) | 0 (0%) | 0.056 |
| 1 | 9 (50%) | 3 (50%) | 6 (100%) |  |
| 2 | 0 (0%) | 1 (17%) | 0 (0%) |  |
| *Reaction description^**^* | | | | |
| Childhood exanthem | 1 (6%) | 0 (0%) | 2 (33%) | 0.19 |
| Immediate diffuse rash | 0 (0%) | 0 (0%) | 0 (0%) |  |
| Diffuse rash > 5-10 years ago | 9 (50%) | 3 (50%) | 3 (50%) | 1.00 |
| Angioedema | 0 (0%) | 0 (0%) | 0 (0%) |  |
| Generalised swelling  (not angioedema) | 0 (0%) | 0 (0%) | 0 (0%) |  |
| Urticaria | 0 (0%) | 0 (0%) | 2 (33%) | 0.069 |
| Anaphylaxis or unexplained collapse | 0 (0%) | 0 (0%) | 0 (0%) |  |
| Unknown reaction > 5-10 years prior | 5 (28%) | 0 (0%) | 0 (0%) | 0.17 |
| Gastrointestinal symptoms | 2 (11%) | 2 (33%) | 1 (17%) | 0.54 |
| Other | 2 (11%) | 1 (17%) | 0 (0%) | 1.00 |
| *Treatment required for reaction* | | | |  |
| Yes | 2 (11%) | 0 (0%) | 1 (17%) | 0.23 |
| No | 9 (50%) | 3 (50%) | 0 (0%) |  |
| Unknown | 7 (39%) | 3 (50%) | 5 (83%) |  |
| *Hospitalized following reaction* | | | |  |
| No | 18 (100%) | 5 (83%) | 5 (83%) | 0.20 |
| Unknown | 0 (0%) | 1 (17%) | 1 (17%) |  |

*IQR: interquartile range, DOC: direct oral challenge,*

*† Other: Autoimmune or connective tissue disorder, human immunodeficiency virus, hemodialysis, end-stage chronic liver disease, splenectomy or prednisolone (or equivalent) use > 10mg/day for 1 month.*

*§ As per previously published AAAT*

** 4 (57%) patients were taking prednisolone (or equivalent corticosteroid) 1-9mg daily; 1 (14%) patient was taking 10-19mg daily; 2 (29%) patients were taking 20-25mg daily*

*** Patients may report more than one reaction description*

# Table S2: Outcomes of 30 immunocompromised hosts undergoing inpatient trimethoprim-sulfamethoxazole direct oral challenge.

| **Outcomes post-DOC** | **Hematological or solid organ malignancy**  **(n=18)** | **Solid organ transplant**  **(n=6)** | **Other*†***  **(n=6)** | **P value** |
| --- | --- | --- | --- | --- |
| Delabeled post-DOC | 18 (100%) | 6 (100%) | 6 (100%) |  |
| Trimethoprim-sulfamethoxazole use post-DOC (at 90-days) | 5 (28%) | 4 (67%) | 3 (50%) | 0.19 |
| *Indication for trimethoprim-sulfamethoxazole use post-DOC (within 90-days)* | | | | |
| Not used | 13 (72%) | 2 (33%) | 3 (50%) | 0.19 |
| Prophylaxis | 3 (17%) | 4 (67%) | 0 (0%) | 0.015 |
| Therapeutic | 2 (11%) | 0 (0%) | 3 (50%) | 0.019 |
| *Indication for therapeutic trimethoprim-sulfamethoxazole (within 90-days)* | | | | |
| Gram-negative bacteraemia | 1 (6%) | 0 (0%) | 2 (33%) | 1.00 |
| Intra-abdominal infection | 0 (0%) | 0 (0%) | 1 (17%) |  |
| Skin and soft tissue infection | 1 (6%) | 0 (0%) | 0 (0%) |  |
| Mortality at 90-days post-DOC | 0 (0%) | 1 (17%) | 1 (17%) | 0.15 |

*DOC: direct oral challenge*

*† Other: Autoimmune or connective tissue disorder, human immunodeficiency virus, hemodialysis, end-stage chronic liver disease, splenectomy or prednisolone (or equivalent) use > 10mg/day for 1 month.*

# Table S3: Estimated healthcare cost savings for utilisation of inpatient trimethoprim-sulfamethoxazole direct oral challenge for immunocompromised patients requiring Pneumocystis jirovecii pneumonia prophylaxis in the Australian setting†

| Drug Costs | | | |
| --- | --- | --- | --- |
| Drug | Dose and administration | Drug costs | Total average costs for 4 weeks |
| Trimethoprim-sulfamethoxazole | 160-800mg orally, daily | $2.62 / 10 tablets | $7.34 |
| Pentamidine | 300mg inhaled, every 4 weeks.  Inpatient admission, via ambulatory care | $413.72 (drug) + $393.26 (hospital ambulatory admission) + $47.25 (catering, administrative) | $854.23 |
| Atovaquone | 1500mg orally, daily | $309.00 / bottle (suspension), not covered under Pharmaceutical Benefits Scheme | $412.00 |
| Direct Drug Cost Savings | | | |
| Drug comparison | 1 month prophylaxis | 6 months prophylaxis |  |
| Trimethoprim-sulfamethoxazole Vs pentamidine | $846.89 | $5081.34 |  |
| Trimethoprim-sulfamethoxazole Vs atovaquone | $404.66 | $2427.96 |  |
| Cost of inpatient trimethoprim-sulfamethoxazole DOC | | | |
| 7/75 inpatients who underwent DOC required PJP prophylaxis, i.e: 1 in 11 patients delabeled | | | |
| Inpatient DOC cost* | 1 patient | 11 patients |  |
|  | $45.00 | $495 |  |
| Healthcare cost savings for delabeling sulfa allergy with trimethoprim-sulfamethoxazole DOC in patients requiring PJP prophylaxis, per delabeled patient | | | |
| Drug comparison | 1 month of prophylaxis | 6 months of prophylaxis | 12 months of prophylaxis |
| Trimethoprim-sulfamethoxazole Vs pentamidine | $31.99 | $416.94 | $878.88 |
| Trimethoprim-sulfamethoxazole Vs atovaquone | $8.21 | $175.72 | $396.45 |

*DOC: direct oral challenge, PJP: pneumocystis jirovecii pneumonia*

**Cost based on previous inpatient penicillin DOC economic analysis in 2020, accounting for inflation^4^*

*†All values are presented in AUD*

# References

1. Trubiano JA, Vogrin S, Chua KYL, et al. Development and Validation of a Penicillin Allergy Clinical Decision Rule. *JAMA Intern Med*. 2020;180:745-752.

2. Waldron JL, Rose M, Vogrin S, et al. Development and Validation of a Sulfa Antibiotic Allergy Clinical Decision Rule. *JAMA Netw Open*. 2023;6:e2316776.

3. Mitri E, Vogrin S, Chua KYL, et al. The long-term sustainability of a pharmacist-led antimicrobial stewardship penicillin allergy delabelling ward round: A prospective cohort study. *CMI Communications*. 2024;1

4. Brusco NK, Bury S, Chua KYL, Vogrin S, Holmes NE, Trubiano JA. Penicillin Allergy Delabeling Program: an exploratory economic evaluation in the Australian context. *Internal medicine journal*. 2023;53:74-83.
